# The hierarchy of root branching order determines bacterial composition, microbial carrying capacity and microbial filtering

**DOI:** 10.1038/s42003-021-01988-4

**Published:** 2021-04-19

**Authors:** William L. King, Caylon F. Yates, Jing Guo, Suzanne M. Fleishman, Ryan V. Trexler, Michela Centinari, Terrence H. Bell, David M. Eissenstat

**Affiliations:** 1 Department of Plant Pathology and Environmental Microbiology, The Pennsylvania State University, University Park, PA 16802 USA; 2 Intercollege Graduate Degree Program in Ecology, The Pennsylvania State University, University Park, PA 16802 USA; 3 Department of Ecosystem Science and Management, The Pennsylvania State University, University Park, PA 16802 USA; 4 Department of Plant Science, The Pennsylvania State University, University Park, PA 16802 USA; 5 Present Address: MOE Key Laboratory of Biosystems Homeostasis and Protection, College of Life Sciences, Zhejiang University, Hangzhou, 310058 China

**Keywords:** Microbial ecology, Plant sciences, Environmental microbiology, Forest ecology

## Abstract

Fine roots vary dramatically in their functions, which range from resource absorption to within-plant resource transport. These differences should alter resource availability to root-associated microorganisms, yet most root microbiome studies involve fine root homogenization. We hypothesized that microbial filtering would be greatest in the most distal roots. To test this, we sampled roots of six temperate tree species from a 23-year-old common garden planting, separating by branching order. Rhizoplane bacterial composition was characterized with 16S rRNA gene sequencing, while bacterial abundance was determined on a subset of trees through flow cytometry. Root order strongly impacted composition across tree species, with absorptive lower order roots exerting the greatest selective pressure. Microbial carrying capacity was higher in absorptive roots in two of three tested tree species. This study indicates lower order roots as the main point of microbial interaction with fine roots, suggesting that root homogenization could mask microbial recruitment signatures.

## Abstract (web summary)

William King, Caylon Yates, and colleagues utilize a 23-year-old common garden experiment to investigate how microbial filtering acts across root branching order. Their results demonstrate that roots of different orders have different microbial assemblages, and point to lower order roots as the main point of microbial interaction with fine roots.

## Introduction

Plants shape the composition of root-adjacent (i.e., rhizosphere/rhizoplane) microorganisms through the release of root exudates and other rhizodeposits^1–5^, which creates a favorable environment for numerous microbial taxa^2,5–8^. Because of this environmental modification, clear differences in microbial composition are often observed between near-root environments and more distal “bulk” soil^6,7,9–11^. Differences between root-adjacent and bulk-soil microbial composition are likely driven by the selective pressure exerted in the root environment, as certain microbial taxa are preferentially recruited^5,12^. Further, the abundance of root exudates and rhizodeposits may facilitate a greater carrying capacity by providing nutrients to house a greater number of cells. Many root-adjacent microorganisms have been shown to influence plant growth and improve resilience to environmental perturbations^13–15^, but root–microbe relationships can also be commensal or deleterious in nature^16–18^. The vast majority of studies that investigate root-adjacent microbial composition have treated fine roots as equal during sampling^6,7,11,19–23^, which does not acknowledge the substantial structural and functional differences observed among different branching orders of the root system^24^. Homogenizing roots with differing functions may mask the microbial signals assigned to absorptive and transportive fine roots, which can skew interpretations of root-driven microbial filtering and recruitment.

Fine root classification has historically relied on size exclusion, in which roots below an arbitrarily chosen size (e.g., <2.0 mm diameter) were considered equivalent^24–26^. This approach can obscure between species comparisons, as it may combine roots with vastly different functions within a plant and different plant species possess disparate fine root morphologies^24,25^. An alternative approach is to classify fine roots according to branching order or functional role. Classification by root order involves designating the most distal roots as root order 1 (R1) and progressively increasing order numbers for root segments that grow closer to the base of the plant^25,27^. Functional classification involves separating fine roots according to their functional role, such as absorptive fine roots (typically includes R1/2) and transport fine roots (includes R4 and above)^24,25^. Structurally, absorptive and transportive roots differ with respect to root hair density, nutrient concentration, and morphology (e.g., development of cork periderm and senescence of root cortex with increasing root order)^25,28,29^. Functionally, lower root orders have greater absorptive capacity, respiration rate, and experience increased mycorrhizal colonization^28,30,31^, whereas higher root orders have greater transport capacity and life spans^24,32,33^.

Most trees form symbiotic relationships with either arbuscular mycorrhizal (AM) or ectomycorrhizal (EM) fungi^34^ and their colonization can vary greatly between absorptive and transportive fine roots^30^. Mycorrhizal fungi enhance the nutrient uptake capacity of their host^35–38^ and trees can specifically exploit these mycorrhizal symbioses to improve nutrient foraging^39^. Although mycorrhizal fungi are believed to interact with some soil bacteria^40–44^, their influence on bacterial composition is likely strongly attributed to leaf litter^45^. Specifically, the leaf litter from AM-associated trees decomposes significantly faster than their EM-associated counterparts^46^ and tree leaf litter can strongly influence the underlying soil properties^45,47^ and, subsequently, the bacterial composition. It is therefore likely that mycorrhizal associations can influence root-associated bacterial filtering and recruitment to some extent.

Redefining how fine roots influence the rhizosphere microbiome also has ecological implications. Differences in fine root function will create unique rhizosphere environments with differing selective pressures on microorganisms. For example, differences in decomposition^48,49^, root morphology^25,29^, water flux^50^, and nutrient content^28,51^ are evident among different root orders. Because of differences in fine root function, the composition of microorganisms associating with different root orders is likely to vary. Homogenizing different root orders, as is done in the vast majority of studies on the root microbiome, may obscure measures of plant investment in microbial selection and of fine-scale microbial filtering. Root homogenization could be particularly problematic when attempting to differentiate the influence of closely related plant genotypes on soil microbial recruitment. Studies have suggested that the phylogenetic signal of microbial recruitment can be relatively subtle^11,23^, but it may be that root homogenization is dulling or entirely obscuring such patterns.

We collected roots from six different temperate tree species that varied widely in mycorrhizal type and root diameter (three AM- and three EM-associating trees) following 23 years of growth in a common garden plantation, and separated these by root order. These tree species were chosen to maximize variations in root diameter and mycorrhizal association, which are both considered key root traits that influence root function^52^. Functionally distinct fine roots were then used to characterize the rhizoplane bacterial composition. We assessed the impact of root order and tree type on bacterial recruitment, as well as bacterial cell counts on a subset of the sampled trees. Our principal hypotheses were as follows: (i) bacterial taxa that have been previously linked to rapid carbon usage would be more prevalent in absorptive fine roots; (ii) absorptive fine roots, which have greater metabolic activity, would exert the greatest selective pressure (microbial filtering) on bacterial recruitment from bulk soil; and (iii) absorptive roots would have a greater microbial carrying capacity relative to transportive fine roots.

## Results

### Different root orders harbor unique microbial assemblages

**Fig. 1:**
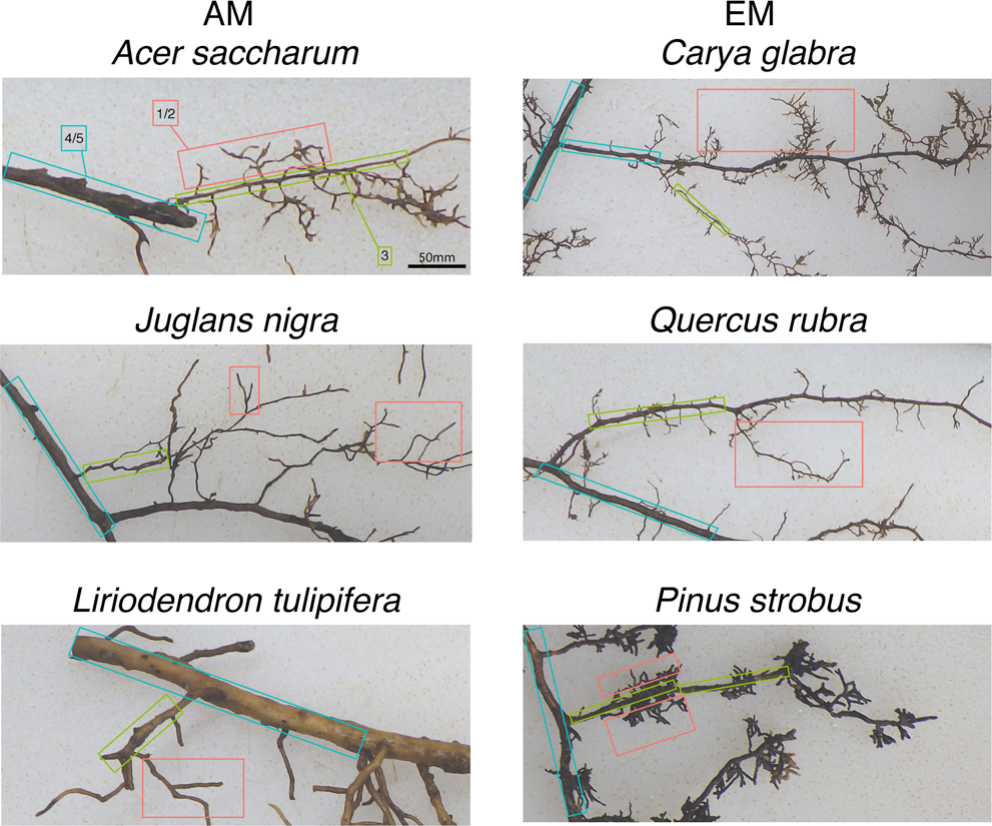
Fine root morphology for six different temperate tree species. Root orders are colored as follows: red is R1/2 (absorptive fine roots), green is R3 (transitionary fine roots), and blue is R4/5 (transportive fine roots). For an enhanced image of R1/2 for each tree species, please see Supplementary Fig. 1. Scale bar is 50 mm.

To identify the influence of root order and mycorrhizal association on bacterial composition, we sampled six tree species (Fig. 1 and Supplementary Fig. 1) from a common garden planting. Root orders were determined according to the topological approach (e.g., see Pregitzer et al.^25^ and McCormack et al.^24^), and for each tree species, we separately collected R1/2 (absorptive), R3 (transitional), and R4/5 (transportive). In general, R1/2 represent newly developed roots with the greatest production of root exudates, R3 is a transitional root type, and R4/5 are typically thicker roots with a well-developed cork periderm that are involved in water and nutrient transport^24,30^.

**Fig. 2:**
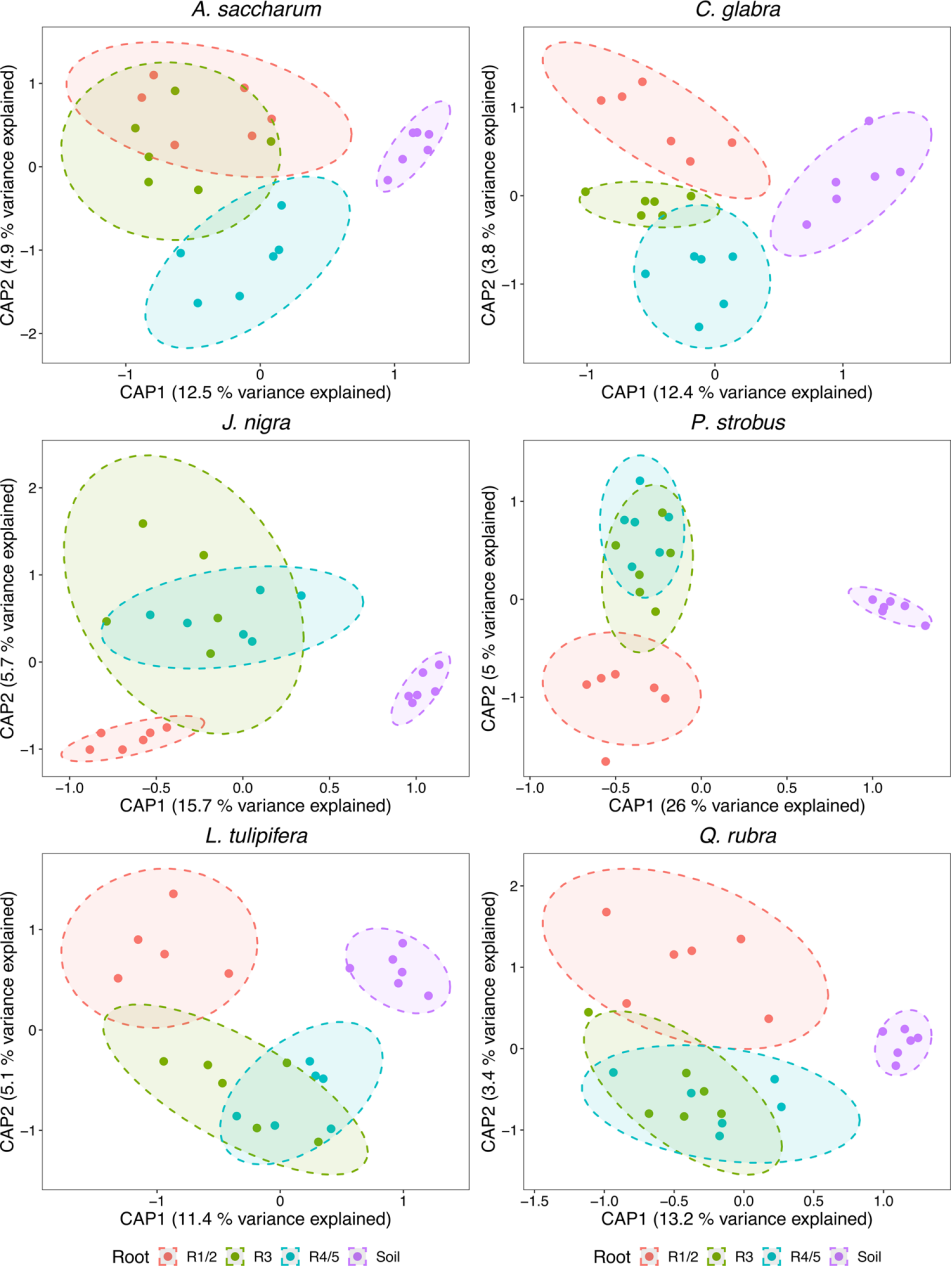
Canonical analysis of principal coordinates (CAP) ordination of individual tree species. Different colors represent different root orders or soil. Ordination was constrained according to root order. 90% Ellipses are shown.

Bacterial composition, based on sequencing of the 16S rRNA gene region, differed significantly by root order (permutational multivariate analysis of variance (PERMANOVA); *F*_2,103_ = 1.4, *R*^2^ = 0.03, *p* = 0.04), which was principally driven by differences between R1/2 and R4/5 (*F*_1,69_ = 1.95, *R*^2^ = 0.03, *p* = 0.01; Supplementary Fig. 2). Significant heterogeneity was observed between different tree species within particular root orders (R1/2: *F*_5,29_ = 2.7, *R*^2^ = 0.32, *p* = 0.001; R3: *F*_5,29_ = 1.9, *R*^2^ = 0.24, *p* = 0.001; R4/5: *F*_5,30_ = 2.2, *R*^2^ = 0.26, *p* = 0.001), whereas differences between tree species appeared to decrease with increasing root order (Supplementary Fig. 3A, B). Differences between root orders were observed within all tree species (Fig. 2 and Table 1) and this was principally driven by differences between R1/2 and R3/R4/5 (Supplementary Table 1).

**Table 1:** PERMANOVA of overall root order comparison per individual tree species.

Overall root order comparison	PERMANOVA result (*F*-value, *R*^2^-value, *p*-value)		
*A*. *saccharum*	*F*_2,15_ = 1.0	*R*^2^ = 0.12	*p* = 0.001
*J*. *nigra*	*F*_2,14_ = 1.3	*R*^2^ = 0.15	*p* = 0.002
*L*. *tulipifera*	*F*_2,14_ = 1.1	*R*^2^ = 0.14	*p* = 0.001
*C*. *glabra*	*F*_2,15_ = 0.9	*R*^2^ = 0.11	*p* = 0.05
*P*. *strobus*	*F*_2,15_ = 1.1	*R*^2^ = 0.13	*p* = 0.001
*Q*. *rubra*	*F*_2,15_ = 0.7	*R*^2^ = 0.09	*p* = 0.001

Soil was not included in each comparison. Provided is the *F*-value (with degrees of freedom as subscript values), *R*^2^ value, and *p*-value.

Soil bacterial compositions significantly differed overall (*F*_5,30_ = 1.5, *R*^2^ = 0.2, *p* = 0.001) but ordination plots indicated this difference was due to high variability in *Carya glabra* soil assemblages (Supplementary Fig. 3A, B). Removal of these samples also removed significance (*F*_4,25_ = 1.2, *R*^2^ = 0.16, *p* = 0.07). In all instances, soil microbial assemblages were distinct from their respective tree root orders, except for *Liriodendron tulipifera* where soil and R4/5 bacterial compositions were not different (Supplementary Table 2).

### Microbial filtering of root bacterial composition decreases with increasing root order

**Fig. 3:**
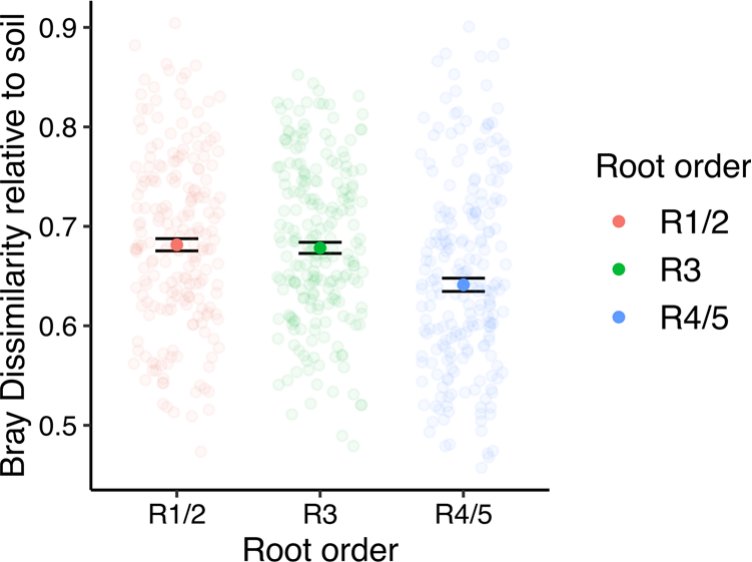
Dot plot of Bray–Curtis dissimilarities of root order samples when compared to soil samples. Data is the Bray dissimilarity median ± SE. Only those distances from within blocks were used to account for the block design. Samples are colored according to root order.

We also aimed to determine whether absorptive roots, which are heavily involved in exchanging materials with microorganisms, were more dissimilar from bulk soil than the higher root orders, which would indicate stronger selection on the root-associated bacterial pool. To assess this, we extracted the Bray–Curtis dissimilarity distances of bulk soil samples to samples from each root order. To mitigate block effects, we only chose dissimilarity distances from within a given block (e.g., Block 1 soil vs. Block 1 Tree R1/2, R3, and R4/5; Fig. 3). Bacterial assemblages associated with R1/2 were the most distinct from bacterial assemblages in bulk soil (Bray dissimilarity median ± SE; 0.681 ± 0.006) followed by R3 (0.678 ± 0.006) and R4/5 (0.64 ± 0.007). We observed significant differences in the similarity of root-associated bacterial composition relative to bulk soil (Kruskal–Wallis test: *H* = 20, 2 d.f., *p* < 0.001), which was principally driven by R4/5 when compared to R1/2 (*H* = 15, 1 d.f., *p* < 0.001) and R3 (*H* = 15, 1 d.f., *p* < 0.001). For individual tree species (Supplementary Fig. 4), differences in root order similarity to soil were detected for *Acer* *saccharum* (*H* = 11, 2 d.f., *p* = 0.004), *Juglans nigra* (*H* = 11, 2 d.f., *p* = 0.004), *L*. *tulipifera* (*H* = 16, 2 d.f., *p* < 0.001), and *C*. *glabra* (*H* = 7, 2 d.f., *p* = 0.04), which was due to increased similarity of soil bacterial compositions and R4/5 relative to R1/2 (*L*. *tulipifera* and *J*. *nigra*) and R3 (*A*. *saccharum*, *J*. *nigra*, and *C*. *glabra*; Supplementary Table 3).

### Root orders display stepwise increases or decreases in the relative abundance of specific bacterial phyla

**Fig. 4:**
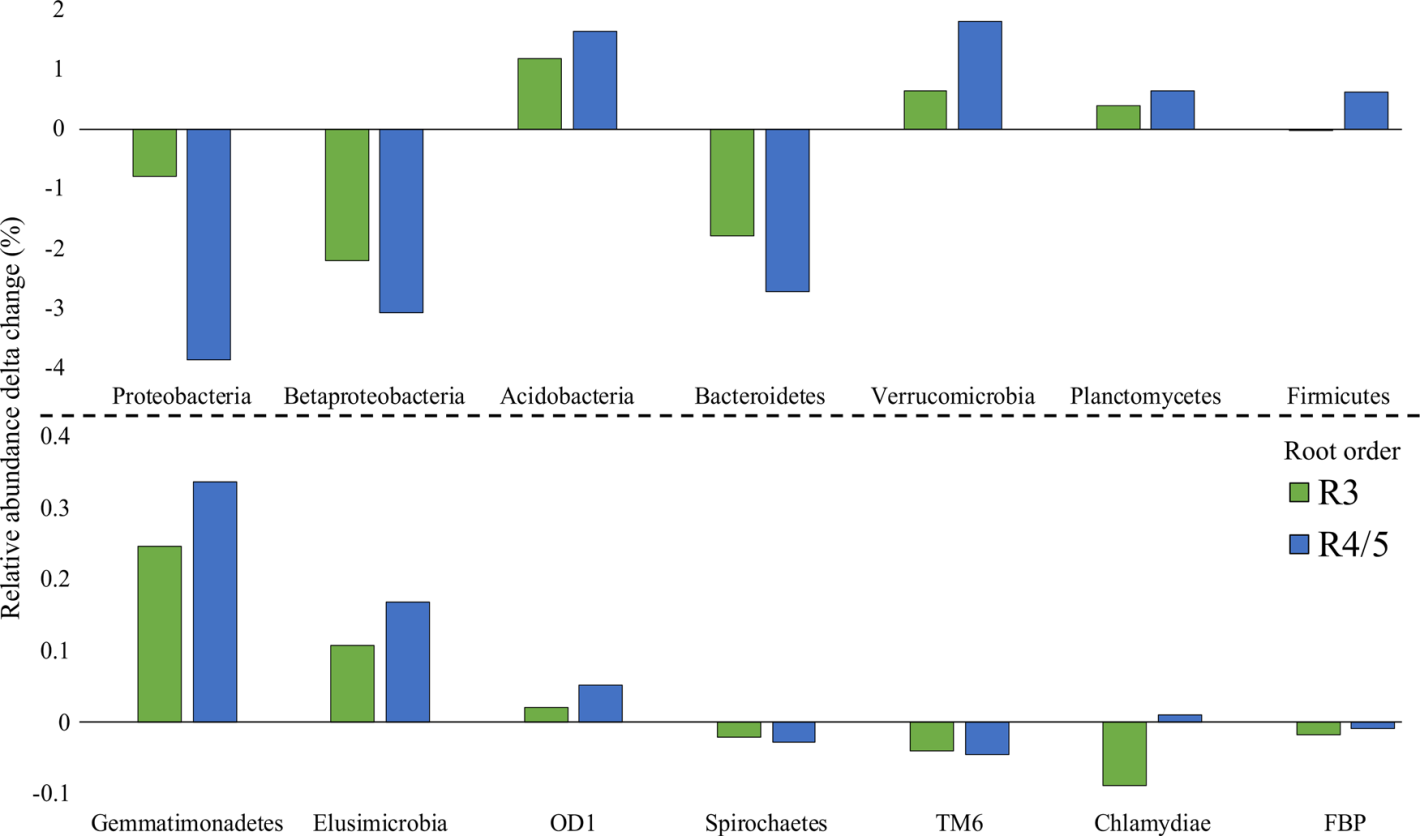
Relative abundance delta changes between root orders for SIMPER identified taxa. All delta changes are relative to R1/2. Columns plotted in the negative direction mean a greater relative abundance in R1/2 relative to either R3 or R4/5. Green columns are R3 and blue columns are R4/5. Only significantly different taxa are shown.

To examine stepwise increases or decreases in taxa between root orders, we performed a similarity percentages (SIMPER) analysis at the phylum level between grouped root orders (Fig. 4). On average, members of the Proteobacteria, Bacteroidetes, Spirochaetes, and TM6 were found to progressively decrease with increasing root order, with the Proteobacteria decrease primarily driven by the Betaproteobacteria (Supplementary Table 4 and Supplementary Fig. 5). In contrast, members of the Acidobacteria, Verrucomicrobia, Plantomycetes, Gemmatimonadetes, Elusimicrobia, OD1, and Firmicutes were found to increase with increasing root order. In addition, we sought to examine conserved sequences across all root samples. We identified four operational taxonomic units (OTUs) as being present in every root sample. These four OTUs were assigned as members of the *Rhodoplanes* (OTU 189524077), *DA101* (of the Verrucomicrobia phylum; OTU 229398176), *Bradyrhizobium* (OTU 374925622), and *Mycobacterium* genera (OTU 814675324), representing an average relative abundance of 0.7%, 2.3%, 3.9%, and 0.6%, respectively.

### Microbial assemblages differ according to mycorrhizal association

When separated according to mycorrhizal association (AM vs. EM), bacterial compositions were distinct when grouping all root orders together (*F*_1,104_ = 8.5, *R*^2^ = 0.08, *p* = 0.001) and when examining root orders separately (R1/2: *F*_1,33_ = 4.2, *R*^2^ = 0.11, *p* = 0.001; R3: *F*_1,33_ = 4.2, *R*^2^ = 0.08, *p* = 0.002; R4/5: *F*_1,34_ = 3.4, *R*^*2*^ = 0.09, *p* = 0.001). Bacterial composition differences between AM- and EM-associating trees were driven by a consistent over-representation of the Acidobacteria in each EM-associating tree root order (R1/2: 16% dissimilarity contribution; R3: 18% dissimilarity contribution; and R4/5: 15% dissimilarity contribution). These patterns confirm our recent observations for absorptive lower-order roots between AM- and EM-associating trees^53^, but also show that these patterns hold true for transitionary and transportive fine roots.

### Absorptive roots house greater numbers of bacteria

**Fig. 5:**
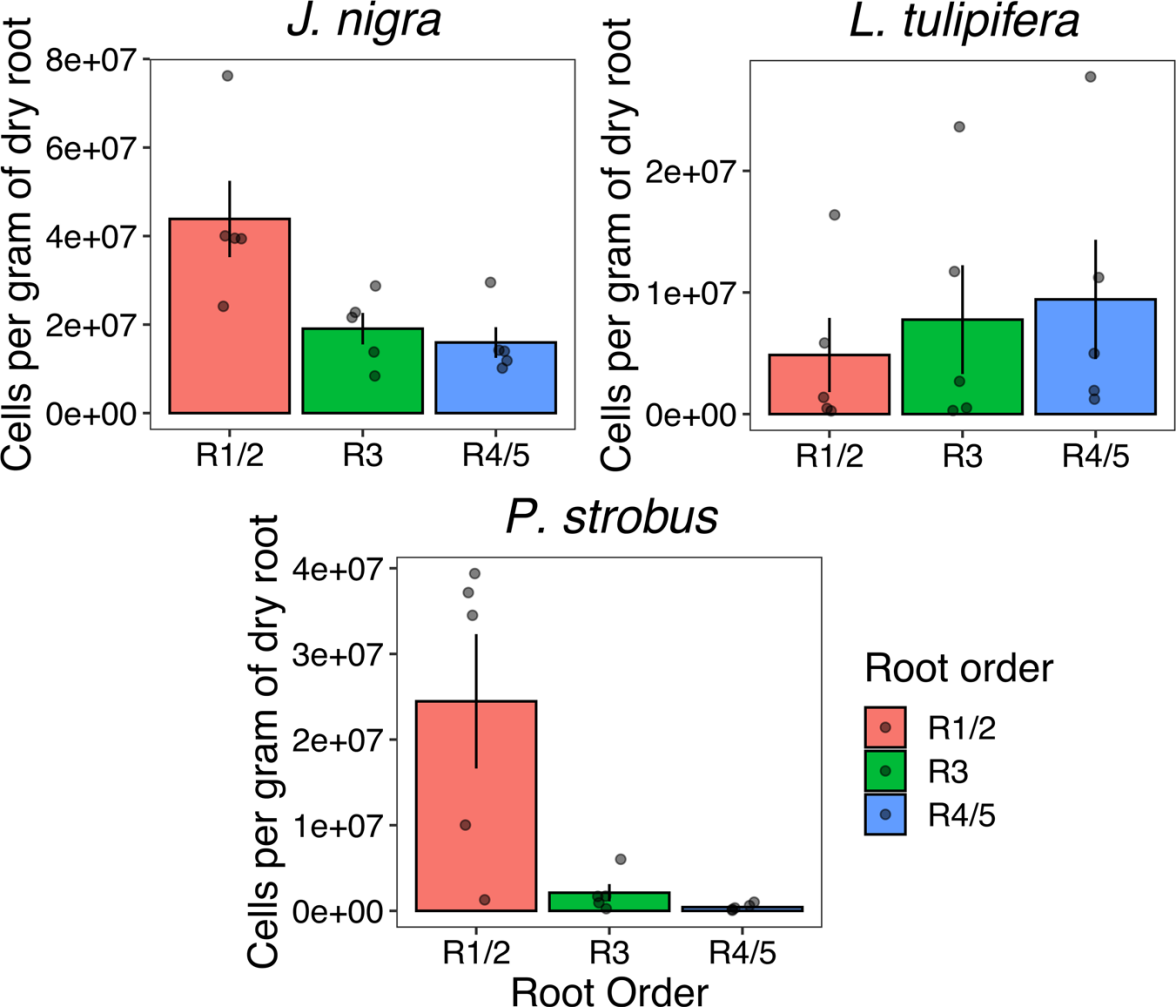
Flow cytometry cell counts plotted for individual trees and root orders. Shown data are the mean and SE. R1/2 was significantly elevated relative to R3 and R4/5 for *J*. *nigra* and *P*. *strobus*.

As absorptive fine roots have more nutrient and water influx relative to transportive fine roots, we expect that the microbial carrying capacity could be higher in the absorptive fine roots. Therefore, to quantify bacterial cell counts between root orders, we performed flow cytometry on three tree species. The tree species *J. nigra*, *L. tulipifera*, and *Pinus strobus* were chosen, as they harbored the most distinct bacterial compositions at their absorptive roots (Supplementary Fig. 3A, B). Overall, bacterial cell counts were significantly higher in *J. nigra* (mean ± SE per gram of dry root; 2.6 × 10^7^ ± 4.5 × 10^6^) relative to both *L. tulipifera* (7.4 × 10^6^ ± 2.3 × 10^6^; Kruskal–Wallis test: *H* = 12.6, 1 d.f., *p* < 0.001) and *P. strobus* (9.0 × 10^6^ ± 3.8 × 10^6^; *H* = 10.6, 1 d.f., *p* = 0.001). When comparing grouped root orders, R1/2 (2.4 × 10^7^ ± 5.7 × 10^6^) had the highest bacterial cell counts when compared to R4/5 (8.6 × 10^6^ ± 2.5 × 10^6^; *H* = 4.6, 1 d.f., *p* = 0.03) with R3 intermediate (9.7 × 10^6^ ± 2.6 × 10^5^). Lack of significance between R1/2 and R3 across all three tree species was primarily driven by highly variable cell counts recorded for *L. tulipifera*. Because of this variability, no differences were observed between individual root orders for *L. tulipifera* (Fig. 5). However, both *J. nigra* and *P. strobus* had significantly elevated cell counts in R1/2 relative to both R3 (*H* = 5.8, 1 d.f., *p* = 0.02; *H* = 3.9, 1 *d.f*., *p* = 0.05; respectively) and R4/5 (*H* = 5.8, 1 d.f., *p* = 0.02; *H* = 5.8, 1 d.f., *p* = 0.009; respectively).

## Discussion

The way in which we define and sample fine roots has broad implications for our understanding of plant-, ecosystem-, and global-scale processes, including nutrient and carbon cycling^24^. Nearly all microbiome-focused studies of root systems involve homogenization of multiple root orders^6,7,11,19–23^, likely because of the laborious nature of sorting fine roots. In root biology studies that do attempt to distinguish between root types, traditional approaches to root classification have relied primarily on size exclusion, wherein all roots below a particular size (e.g., 2.0 mm diameter) were considered equivalent^25^. However, such size-exclusion approaches do not account for substantial cross-species variability in root morphology or the considerable variability in functional roles within the branches of a fine root system^24,25^. For example, a 2.0 mm diameter classification would include all fine roots up to the fourth root order for *L*. *tulipifera*^24^.

**Fig. 6:**
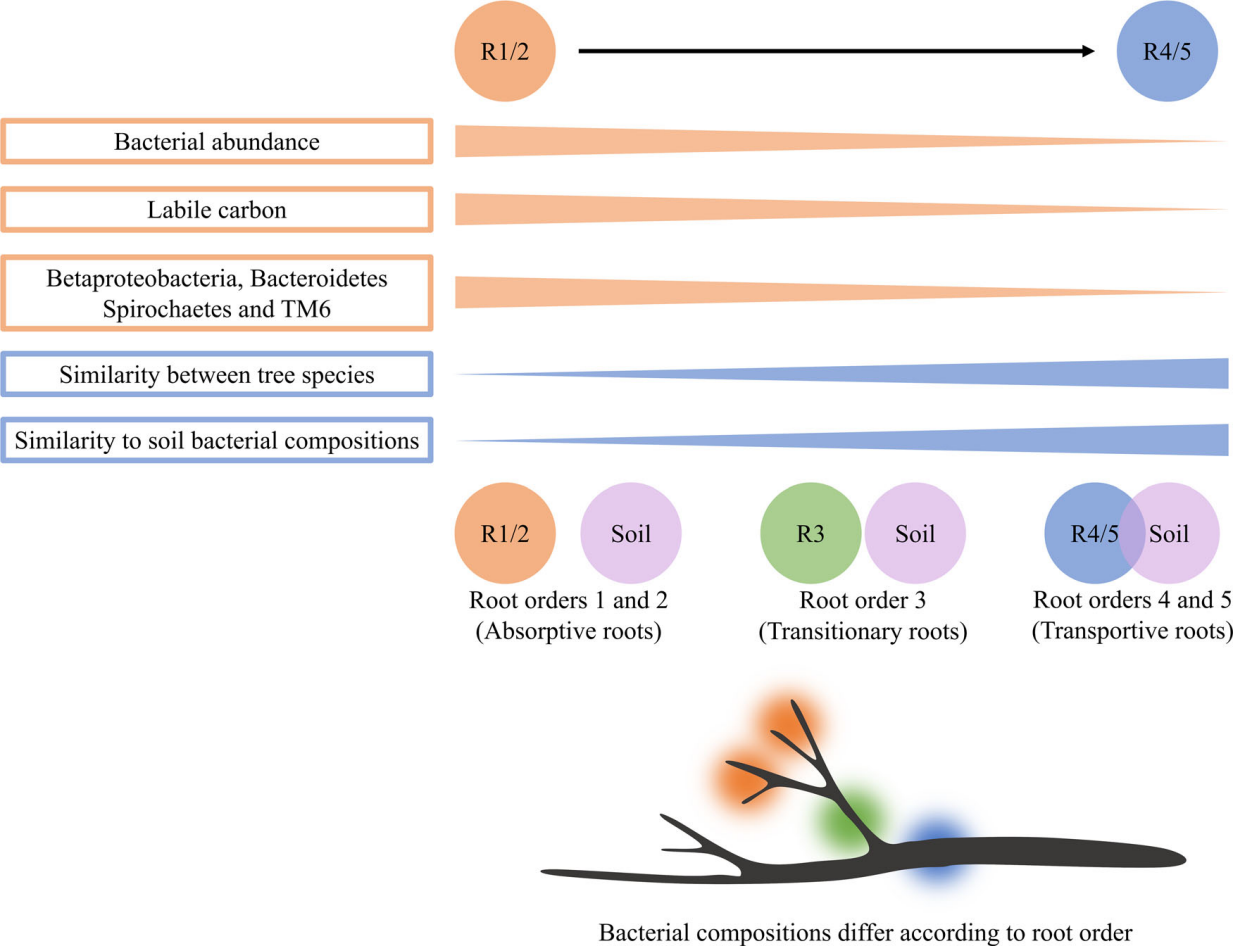
Conceptual framework of differences in bacterial composition by root order. The composition of root-associated bacteria was most distinct from bulk soil in the lowest root orders, where bacterial abundance was also typically higher. Taxa associated with carbon mineralization were found in greater relative abundance in root orders 1 and 2, suggesting that copiotrophic phyla preferentially colonize absorptive fine roots with greater nutrient flux. R1/2 is colored orange, R3 is green, and R4/5 is blue.

Our data demonstrate clear differences in bacterial composition according to root order for six phylogenetically diverse tree species, which vary widely in fine root traits (Fig. 6). We expect that these differences are likely driven by differences in the functional role of each root order. In newly developed absorptive roots, we expect higher respiration and increased flux of labile carbon, water, and nutrients relative to the longer lived but less absorptive transportive fine roots^24,32^. Supporting this, we observed a greater relative abundance of higher taxa associated with efficient carbon mineralization in absorptive roots, namely the Betaproteobacteria and Bacteroidetes^54^, and an under-representation of the Acidobacteria, which have been shown to correlate negatively with carbon mineralization^54^. Recent developments for our understanding of fine root dynamics in soil have identified differences in fine root morphology and turnover rates according to soil depth, and seasonal effects on fine root production, biomass, necromass, and mortality^55^. Further, nonlinear relationships between root diameter, tissue density, and nitrogen concentration have been recently identified for woody plant species^56^. As our understanding of fine root dynamics improves so too will our understanding of the conditions required for microbial colonization and proliferation. For example, and depending on the season of sampling, higher rates of fine root mortality could shift microbial dynamics towards decomposers, which could alter compositional differences between branching root orders.

As absorptive roots are a hotspot for nutrient flux^31,57^, we expected a greater microbial carrying capacity in these microenvironments. We used flow cytometry to quantify bacterial cells from three of the six sampled trees, and results from two of these provided a strong support for our expectation. For *J*. *nigra* and *P*. *strobus*, R1/2 harbored the greatest bacterial abundance relative to R3 and R4/5. The bacterial abundance was almost an order of magnitude greater for *P. strobus* and nearly three times greater for *J. nigra* when compared to R4/5. This suggests that absorptive fine roots, at least in some species, have a greater microbial carrying capacity relative to transitionary and transportive fine roots. The increased microbial carrying capacity is likely directly related to the greater nutrient flux in absorptive fine roots^31,57^ and corresponds to increases in taxa known for their copiotrophic lifestyles (e.g., Betaproteobacteria and Bacteroidetes)^54^. These data further highlight the need for differentiating fine roots according to their functional roles, as root homogenization can combine fine roots with extremely disproportionate bacterial abundance (e.g., fine root bacterial abundance as almost an order of magnitude different for *P. strobus*).

With increasing root order, root-associated microbial composition converged on those observed in bulk soil. This suggests that transportive fine roots exert less selective pressure on microorganisms than absorptive fine roots, which is also supported by our observation of increased cell abundance in the absorptive fine roots of two of three tree tested tree species. Selective bacterial recruitment by absorptive roots could be driven by root exudates and rhizodeposits^4^, which are known to influence microbial community composition^3,4,22^. Methods for root sampling can therefore significantly impact the composition of observed microorganisms and our data suggest that homogenization or size exclusion could dull patterns of microbial selection. This has important impacts on our understanding of plant-driven microbial recruitment and, in particular, our ability to detect differences in microbial selection across closely related plant genotypes. Although our data demonstrate that homogenization of different root orders can dull microbial signals and lead to erroneous interpretations of microbial data, we did not include a “homogenization” control as a comparative sample. The magnitude of error from homogenization is difficult to predict and will ultimately be strongly influenced by the surface area contribution by each root order in the homogenized sample. In a typical four to five branching root system, absorptive fine roots contribute a greater proportion to root length and quantity (number of segments), whereas transportive fine roots contribute a greater proportion to root mass^25,58^. The proportionally higher abundances of absorptive fine roots may diminish errors when homogenizing roots, but this would be species specific. Regardless, we observed differences in microbial patterns across root orders and these microbial signals would be diluted by combining multiple root orders into one sample.

In addition to those bacterial composition differences observed between different fine roots, we also identified broad patterns separating microbial assemblages for AM- and EM-associating trees for each individual root order. In support of our data, differences between bacterial compositions for AM- and EM-associating trees for absorptive fine roots have been recently noted^53^ and our data highlight that this pattern is consistent for higher-order roots. Close associations between mycorrhizal fungi and particular bacterial taxa have been previously noted^40–44,59,60^, which could explain the bacterial composition separation observed in this study. We found that members of the Acidobacteria were consistently over-represented in EM-associating plants relative to AM-associating plants for each root order. In agreement with this finding, Acidobacteria are notable for their ability to suppress AM fungi activity^41^.

Although we observed clear differences in bacterial composition, microbial filtering, and microbial carrying capacity according to branching root order, caution should be applied when generalizing these patterns to natural conditions. Our experimental design used a 23-year-old common garden planting, and while soil conditions were homogenous at the time of planting^61^, these conditions may not necessarily reflect the response of plants growing in natural conditions. Certainly, our common garden planting would be considered a partly controlled environment and differences in root traits, such as fine‐root tissue density, have been identified previously when compared to natural settings^62^. However, root trait similarities between common garden plantings and natural conditions have been observed for nitrogen concentration, root diameter, and root length^62^.

Differing approaches to fine root sampling can dramatically alter our view of belowground processes. In recent years, assessments of root-associated microbiomes have dramatically increased in number and most of these rely on homogenization of root clusters in our systems. This study identified significant differences in bacterial composition between fine root orders, with often significantly elevated microbial cell counts in absorptive fine roots relative to transportive fine roots. These data highlight differences in microbial carrying capacity, and root-driven microbial filtering and selective pressure of bacterial composition. These differences are notable for experimental designs which homogenize fine roots with different functionality, as this can mask the observable microbial patterns, particularly if the microbial signal is subtle. Accurate classification of fine roots according to their functional roles has ecological implications for a number of different fields, particularly microbiology. Here we have shown differences in microbial carrying capacity, microbial filtering and selective pressure, and bacterial compositions for functionally discrete fine roots. Therefore, future studies aiming to characterize plant–microbe–environment interactions will need to account for varying fine root orders, to ensure subtle microbial signals are not masked by homogenization and to avoid spurious interpretations of their microbial patterns.

## Methods

### Study site

The experimental site was a common garden forest located at the Russell E. Larson Agricultural Research Center in central Pennsylvania (40°42′ N, 77°57′ W), which has previously been described in detail^61^. Briefly, this common garden forest contains 16 different tree species, which were planted as 1-year-old seedlings in 1996 and was constructed with a randomized complete block design. Soil conditions were homogenous throughout the site prior to seedling planting^61^. For this study, we selected three AM-associating (*A. saccharum*, *J. nigra*, and *L. tulipifera*) and EM-associating (*C. glabra, Quercus rubra*, and *P. strobus*) tree species that represented a variety of taxonomic groups and root traits.

### Sample collection

Roots and bulk soil were sampled in 2018 on 3 July (blocks 1–4) and 13 July (blocks 5 and 6). Sampling was performed on two separate days because of the labor-intensive nature of sampling. Roots were collected using a spading fork and gently shaken to remove loosely adherent soil. Root order was determined by the topological approach (e.g., see Pregitzer et al.^25^ and McCormack et al.^24^). For each tree species, we collected R1/2 (absorptive), R3 (transitional), and R4/5 (transportive) from two root clusters for each single-species plot from each of the six blocks. Samples were transported to the laboratory on ice and stored at −20 °C until needed.

Soil organic matter was uniform across the common garden plantation, with differences in pH observed for some tree species^53^. Absorptive root diameter did not explain differences in soil properties between species^53^. Measures of root branching ratio, branching intensity, and root diameter are provided in Supplementary Table 5.

### DNA extraction and sequencing

Sampled roots were transferred into a 1.5 ml NucleoSpin^®^ bead tube and 700 µL of lysis buffer (SL1) was added. Samples were sonicated for 5 min (Branson Bransonic Ultrasonic Bath) and the roots were subsequently removed. DNA was extracted from bulk soil samples and the sonicated solutions using a NucleoSpin^®^ 96 soil DNA extraction kit (catalog: 740787.2) according to the manufacturer’s instructions.

Extracted DNA were amplified using the 515F^63^ and 806R^64^ primer pair, as previously described^65^. Briefly, PCR ingredients were as follows: 8 μl of 5Prime HotMasterMix (Quanta BioSciences, Inc.), 1 μl template DNA, 1 μl of each primer (10 μM), and 9 μl molecular grade water, for a final volume of 20 μl. PCR cycling conditions were as follows: 94 °C for 3 min, 25 cycles of 94 °C for 30 s, 55 °C for 30 s, and 72 °C for 45 s, and a final elongation step for 10 min at 72 °C. Amplicons were then purified with Mag-Bind TotalPure NGS clean-up beads (Omega Bio-Tek). A second PCR was used to add Illumina adapters and indexing barcodes to the purified amplicons. The second PCR ingredients were as follows: 5 μl of cleaned PCR product, 12.5 μl of 5Prime HotMasterMix, 2.5 μl of water, and 2.5 μl of each index primer (10 μM), for a final volume of 25 μl. The second PCR conditions were as follows: 98 °C for 1 min, 8 cycles of 98 °C for 15 s, 55 °C for 30 s, and 72 °C for 20 s, followed by a final elongation step for 5 min at 72 °C.

Barcoded and indexed amplicons were normalized using a SequalPrep Normalization Plate Kit (Invitrogen) and pooled. Pooled amplicons were concentrated using a Savant SpeedVac (Thermo Scientific) at 50 °C for 3 h. Concentrated DNA was run in a 1.2% agarose gel and the expected band was excised and extracted using a PureLink Quick Gel Extraction Kit (Invitrogen). Purified pooled amplicons were sent to the Cornell University Biotechnology Resource Center Genomics Facility to be sequenced on the Illumina MiSeq platform (2 × 250 cycle v2 kit). Raw data files in FASTQ format were deposited in the NCBI sequence read archive under Bioproject number PRJNA639455. In total, we characterized the bacterial composition of 108 root samples and 36 bulk soil samples targeting the 16S rRNA V4 region.

### Sequencing analysis

Raw fastq data were processed with Mothur^66^ (version 1.36) and QIIME^67^ (version 1.9.1). In Mothur, paired end reads were merged with *make.contigs*. trimmed with *trim.seqs* (pdiffs = 2) and singletons removed using *split.abund*. In QIIME, joined reads were dereplicated, clustered at the 97% threshold, and chimeras removed using USEARCH^68^. Taxonomy was assigned in Mothur against the GreenGenes^69^ (version 13.8.99) database. Processed data were then imported into the R statistical environment and further cleaned. First, OTUs assigned as mitochondria, archaea, chloroplasts, and unclassified phyla were removed. Data were then rarefied to 5427 counts per sample, which was chosen to preclude the inclusion of two samples with low counts (38 and 191 counts). Data were then transformed compositionally (relative abundance) and used to produce a Phyloseq^70^ object for further analyses.

### Flow cytometry

To quantify microbial cell counts between tree species and root orders, we quantified cells in the rhizoplane and rhizoplane soil using flow cytometry. We chose three tree species *J. nigra*, *L. tulipifera*, and *P. strobus*, as they harbored the most distinct bacterial compositions at their absorptive roots (Supplementary Fig. 3A, B). Fresh root samples were collected in the following year and were stored at 4 °C prior to cell extraction from the rhizoplane and rhizoplane soil. Equal mass of root samples for each root order were visually assessed and transferred into a 1.5 ml tube. The cell extraction was performed as previously described^71^. Briefly, 700 μl of sterile NaCl 0.85% solution was added into each tube and the roots were sonicated (Branson Bransonic Ultrasonic Bath) for 5 min. Sonication removed the rhizoplane microbes from the root surface into the NaCl solution. Roots were then removed from the sample and each sample was homogenized by vortexing for 5 min at full speed. The homogenized suspension was centrifuged at 130 × *g* for 5 min, to exclude the large soil particles. The supernatant was then filtered at 40 μm, to remove particles for the further analyses. From this filtered solution, we took a 250 μl aliquot and stained it with 1 μL of SYBR® Green I (10,000× in dimethyl sulfoxide; Life Technologies). All samples were incubated at room temperature for 15 min in the dark. Absolute counts were achieved by using a Flow-Count Fluorosphere according to the manufacturer’s instructions. Cells were quantified using a MACSQuant Vyb flow cytometer (Miltenyi) with a 488 nm blue laser and a 525/50 nm channel.

### Statistics and reproducibility

To compare bacterial assemblages between root orders and differing fungal mycorrhizal associated trees, a principal coordinates analysis (PCoA) with a Bray–Curtis dissimilarity index was used. To examine root orders within tree species, a constrained canonical analysis of principal coordinates (CAP) was used with a Bray–Curtis dissimilarity index. PCoA and CAP were performed using the ordinate function in the Phyloseq package^70^. Patterns elucidated by the PCoA were statistically tested using Adonis (PERMANOVA) from the vegan package^72^ with 999 permutations. To identify taxa driving the difference between groups, a SIMPER analysis was used from the vegan package with a Bray–Curtis dissimilarity index. Data were summarized at different taxonomic levels using the MicrobeR package^73^. Comparisons of flow cytometry cell count data were performed using a Kruskal–Wallis test from the stats package^74^. To compare Bray–Curtis dissimilarities of root orders relative to soil, a Kruskal–Wallis test from the stats package was used, followed by a Dunnett’s post hoc test. All statistical tests were performed in the R statistical environment^74^. Sample sizes were as follows: six blocks, six tree species (three AM- and three EM-associating) per block, and three root orders or bulk soil from each tree species (six replicates for each root order or bulk soil per tree species). After rarefaction, two replicates were removed (*L*. *tulipifera* R1/2 and *J*. *nigra* R3). For flow cytometry cell counts, each root order for each tree species had five replicates.

### Reporting summary

Further information on research design is available in the Nature Research Reporting Summary linked to this article.

## Supplementary information

Supplementary Information

Reporting Summary

Supplementary Data 1

## Data Availability

Raw data files in FASTQ format were deposited in the NCBI sequence read archive under Bioproject number PRJNA639455 and supporting data for this manuscript can be found in Supplementary Data 1.
