# Supplementary material for: The hierarchy of root branching order determines bacterial composition, microbial carrying capacity and microbial filtering
Source: Commun Biol. 2021 Apr 19;4:483. doi: 10.1038/s42003-021-01988-4 (PMC8055976; doi:10.1038/s42003-021-01988-4)
Supplement: Supplementary file 1 — Supplementary Information [file 42003_2021_1988_MOESM1_ESM.pdf]

The hierarchy of root branching order determines bacterial composition, microbial carrying capacity and microbial filtering

William L King[1†], Caylon F Yates[1,2†], Jing Guo[3#], Suzanne M Fleishman[2,3,4], Ryan V Trexler[1], Michela Centinari[2,4], Terrence H Bell[1,2*], David M Eissenstat[2,3*]

[1]Department of Plant Pathology and Environmental Microbiology, The Pennsylvania State University, University Park, PA, 16802, USA

[2]Intercollege Graduate Degree Program in Ecology, The Pennsylvania State University, University Park, PA, 16802, USA

[3]Department of Ecosystem Science and Management, The Pennsylvania State University, University Park, PA, 16802, USA

[4]Department of Plant Science, The Pennsylvania State University, University Park, PA, 16802, USA

[#]*Present address:* MOE Key Laboratory of Biosystems Homeostasis & Protection, College of Life Sciences, Zhejiang University, Hangzhou, 310058, China

[†]These authors contributed equally

[*]Correspondence:
THB: thb15@psu.edu
DME: dme9@psu.edu

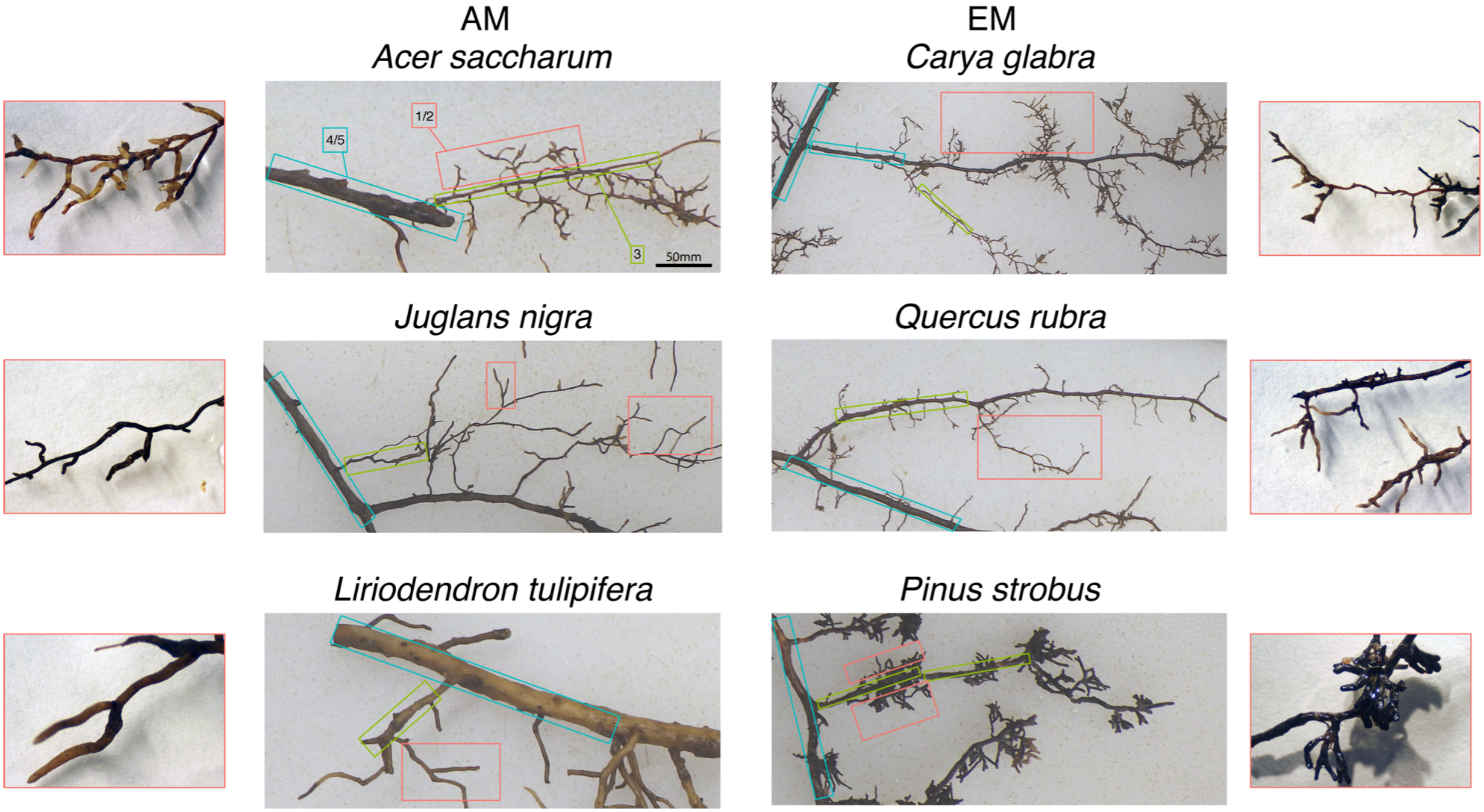

Supplementary Figure 1: Fine root morphology for six different temperate tree species. Root orders are colored as follows: Red is R1/2 (absorptive fine roots), green is R3 (transitionary fine roots) and blue is R4/5 (transportive fine roots). Scale bar is 50 mm. An enhanced image of R1/2 is provided next to their respective tree species.

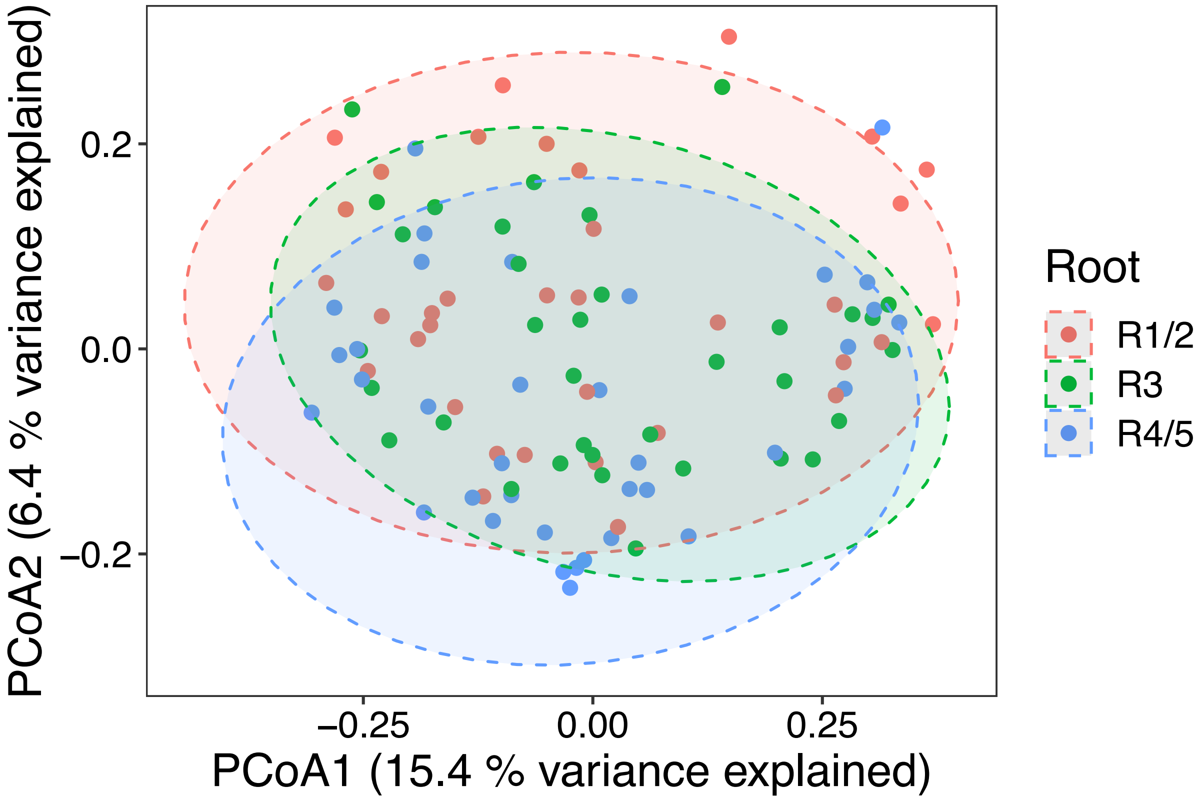

Supplementary Figure 2: PCoA ordination plots for grouped root orders. Samples are colored according to root order and ellipses represent 90 % of the data.

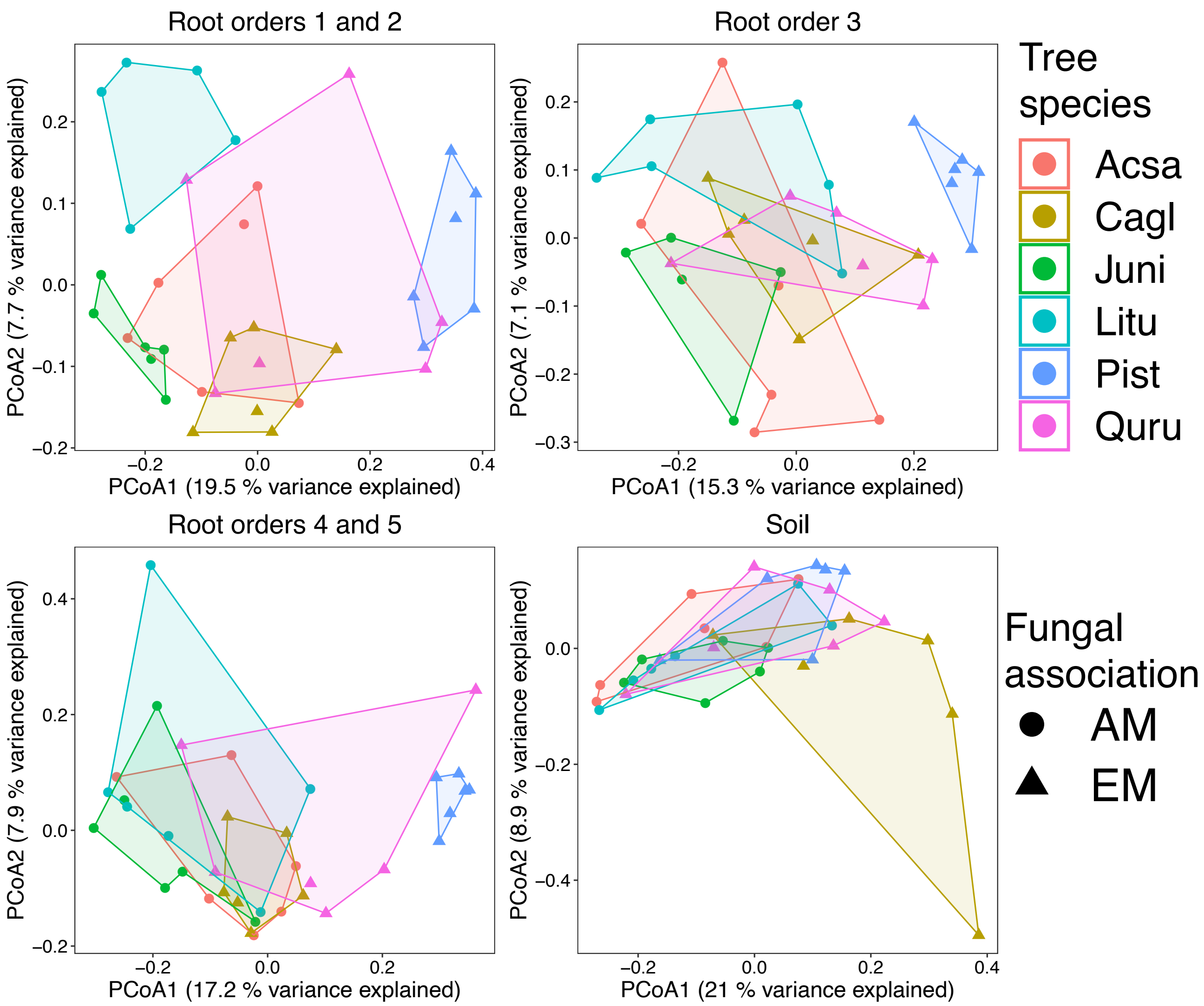

Supplementary Figure 3A: PCoA ordination plots for individual tree species per root order. Different tree species are represented by different colors. Arbuscular (AM) and ectomycorrhizal (EM) fungal associations are represented as circles and triangles, respectively. Species abbreviations are as follows: Acsa is *A. saccharum*, Cagl is *C. glabra*, Juni is *J. nigra*, Litu is *L. tulipifera*, Pist is *P. strobus* and Quru is *Q. rubra*.

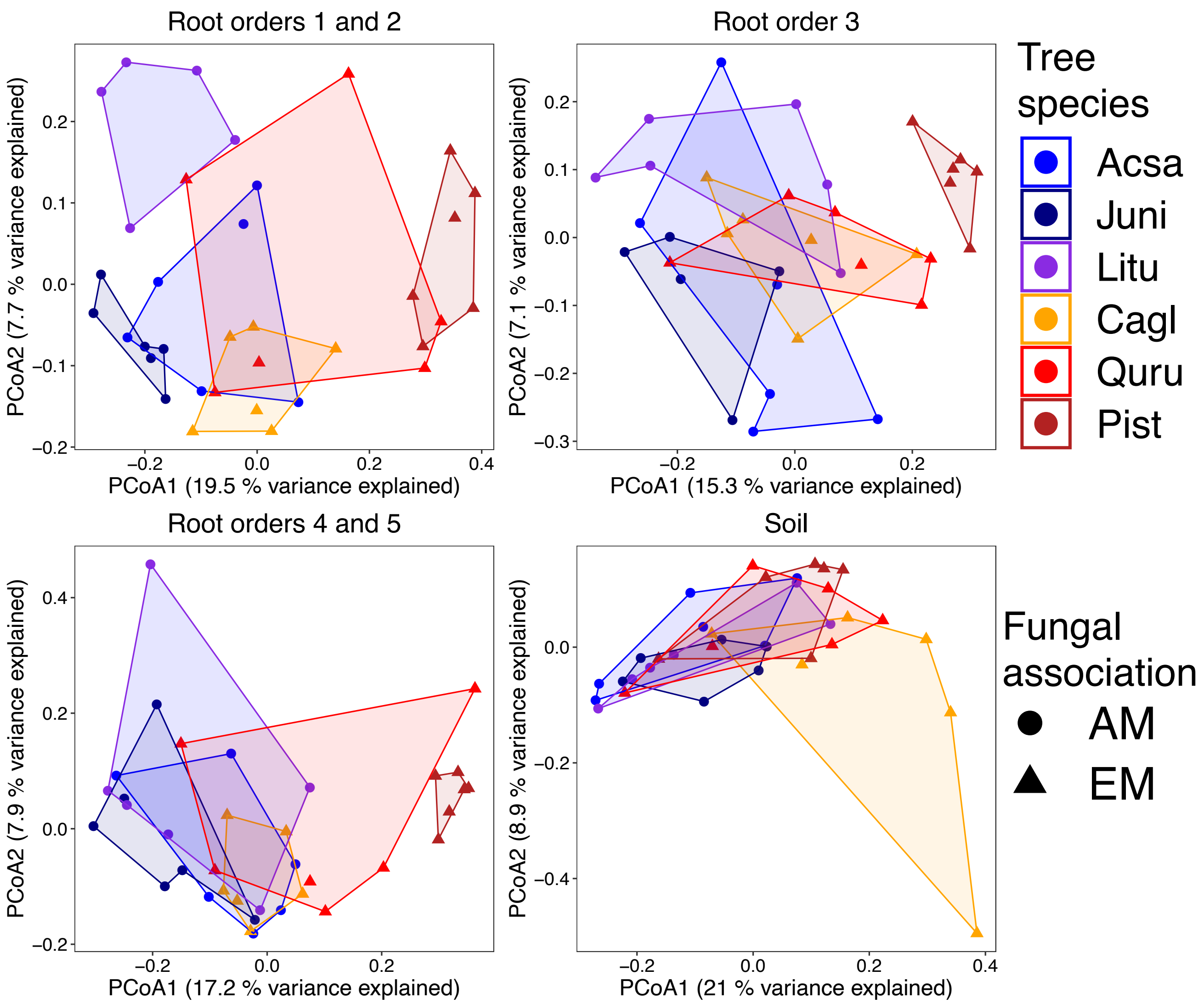

Supplementary Figure 3B: PCoA ordination plots for individual tree species per root order. Arbuscular (AM) fungal associations are represented as circles and are colored on the blue spectrum. Ectomycorrhizal (EM) fungal associations are represented as triangles and are colored on the red spectrum. Species abbreviations are as follows: Acsa is *A. saccharum*, Juni is *J. nigra*, Litu is *L. tulipifera*, Cagl is *C. glabra*, Quru is *Q. rubra* and Pist is *P. strobus*.

Supplementary Table 1: Adonis for individual root orders within individual trees. Degrees of freedom for all comparisons was 1. Provided is the R^2 and p-value.

Tree species	R1/2 vs R4/5	R1/2 vs R3	R3 vs R4/5
A. saccharum	$R^2 = 0.11, p = 0.03$	$R^2 = 0.08, p = 0.03$	$R^2 = 0.09, p = 0.04$
J. nigra	$R^2 = 0.14, p = 0.05$	$R^2 = 0.12, p = 0.03$	$R^2 = 0.09, p = 0.4$
L. tulipifera	$R^2 = 0.14, p = 0.03$	$R^2 = 0.11, p = 0.03$	$R^2 = 0.07, p = 0.09$
C. glabra	$R^2 = 0.09, p = 0.1$	$R^2 = 0.08, p = 0.4$	$R^2 = 0.08, p = 0.08$
P. strobus	$R^2 = 0.12, p = 0.03$	$R^2 = 0.11, p = 0.03$	$R^2 = 0.07, p = 0.03$
Q. rubra	$R^2 = 0.07, p = 0.06$	$R^2 = 0.07, p = 0.03$	$R^2 = 0.06, p = 0.03$

Supplementary Table 2: Statistical comparisons of soil microbial communities to their respective plant root orders. Data is displayed as F-value; R^2; p-value. Degrees of freedom for all comparisons was either (1,10) or (1,9).

	Tree species	Root order		
		R1/2	R3	R4/5
Soil	*A. saccharum*	3.0; 0.23; 0.006	2.9; 0.22; 0.002	2.56; 0.20; 0.007
	C. glabra	2.5; 0.20; 0.001	2.5; 0.20; 0.004	1.9; 0.16; 0.004
	J. nigra	4.8; 0.33; 0.002	2.8; 0.24; 0.005	2.5; 0.20; 0.003
	L. tulipifera	2.8; 0.24; 0.002	2.0; 0.17; 0.002	No significance
	P. strobus	6.5; 0.40; 0.003	5.6; 0.36; 0.003	5.3; 0.35; 0.005
	Q. rubra	2.6; 0.21; 0.004	3.2; 0.24; 0.003	2.2; 0.18; 0.003

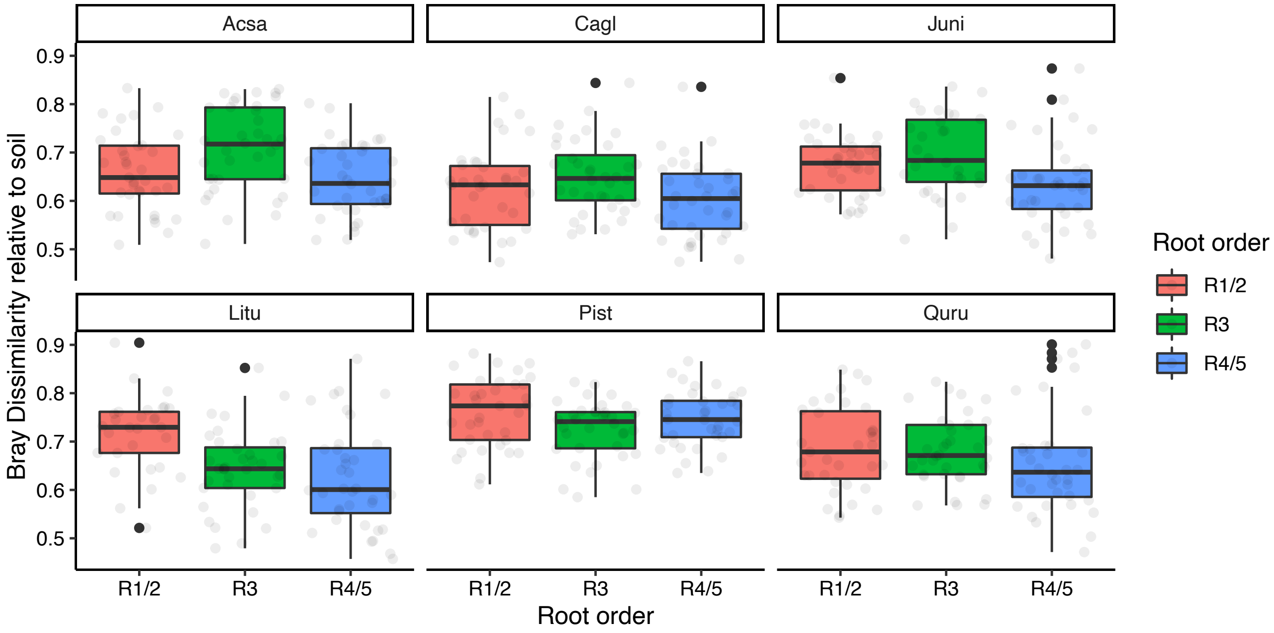

Supplementary Figure 4: Boxplot of Bray-Curtis dissimilarities of root order samples when compared to soil samples for individual tree species. Only those distances from within blocks were used to account for the block design. Samples are colored according to root order. Species abbreviations are as follows: Acsa is *A. saccharum*, Cagl is *C. glabra*, Juni is *J. nigra*, Litu is *L. tulipifera*, Pist is *P. strobus* and Quru is *Q. rubra*. Centre line is median. Outliers are black dots. Data distribution is grey dots. Upper and lower quartiles are 75 and 25 percentiles, respectively.

Supplementary Table 3: Bray-Curtis dissimilarities comparisons for soil versus root orders. Comparisons were extracted within blocks. Shown data is from Dunnett's test and the Z statistic and adjusted p-value (q-value) are shown. NS means not significant.

Tree species	R1/2 vs R4/5	R1/2 vs R3	R3 vs R4/5
A. saccharum	NS	$Z = 2.4$, $q = 0.03$	$Z = 3.2$, $q = 0.005$
J. nigra	$Z = 2.4$, $q = 0.03$	NS	$Z = 3.1$, $q = 0.005$
L. tulipifera	$Z = 3.8$, $q < 0.001$	$Z = 3.0$, $q = 0.004$	NS
C. glabra	NS	NS	$Z = 2.6$, $q = 0.03$

Supplementary Table 4: Taxa with significantly different relative abundance between different root orders. Three pairs of SIMPER analyses at the Phylum level were performed. Because of the high relative abundance of the Proteobacteria, a SIMPER analysis was also performed at the class level to identify the class driving the stepwise decrease. Average relative abundance is provided for each root order.

Taxa	R1/2 (%)	R3 (%)	R4/5 (%)
Proteobacteria	48.0	47.2	44.1
Betaproteobacteria	10.1	7.9	7.0
Acidobacteria	12.8	14.0	14.5
Bacteroidetes	8.8	7.0	6.1
Verrucomicrobia	8.9	9.5	10.7
Planctomycetes	2.6	3.0	3.2
Gemmatimonadetes	0.78	1.0	1.1
Elusimicrobia	0.16	0.26	0.32
OD1	0.08	0.11	0.14
Spirochaetes	0.05	0.03	0.02
TM6	0.36	0.32	0.31
Chlamydiae	0.33	0.24	0.34
FBP	0.03	0.01	0.02
Firmicutes	0.70	1.01	1.01

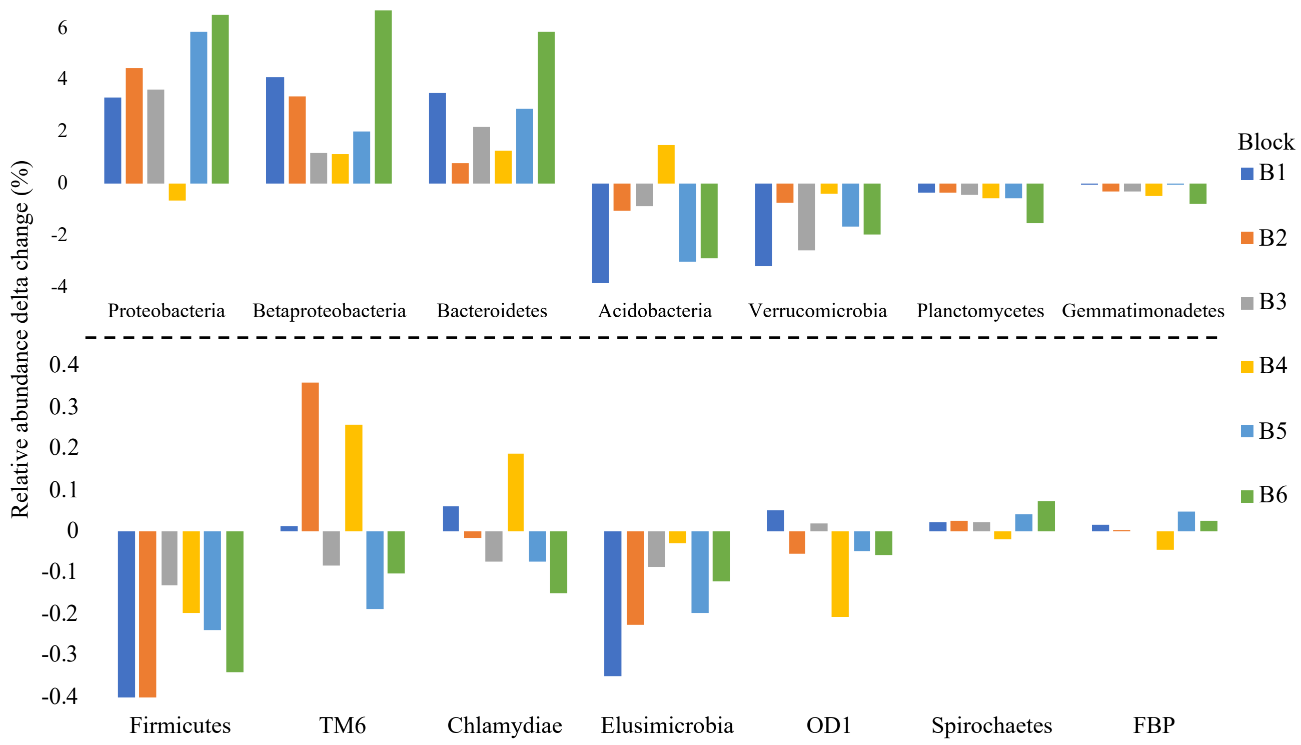

Supplementary Figure 5: Relative abundance delta changes between R1/2 and R4/5 for SIMPER identified taxa. Columns plotted in the positive direction mean a greater relative abundance in R1/2 relative to R4/5. Column colors denote the six individual blocks.

Supplementary Table 5: Root anatomical measurements for absorptive and intermediary fine roots. Root branching ratio and intensity were measured and calculated from ingrowth core data. Root diameter for absorptive fine roots was derived from McCormack et al. 2012[1]. An average of branching ratio and intensity is provided from eight replicates.

	Branching ratio		Branching intensity			Diameter (mm)
Species	1st/2nd	2nd/3rd	#1st /cm	#2nd /cm	#3rd /cm	1st and 2nd
A. saccharum	5.75	4.05	2.14	0.40	0.07	0.30
C. glabra	5.10	3.35	5.57	1.13	0.35	0.22
J. nigra	5.52	2.50	1.08	0.20	0.03	0.30
L. tulipifera	4.18	0.31	0.87	0.16	0.02	0.64
P. strobus	6.25	0.81	2.50	0.51	0.08	0.31
Q. rubra	4.51	3.12	3.62	0.83	0.23	0.23

1 McCormack, M., Adams, T. S., Smithwick, E. A. H. & Eissenstat, D. M. Predicting fine root lifespan from plant functional traits in temperate trees. *New Phytologist* **195**, 823-831, doi:10.1111/j.1469-8137.2012.04198.x (2012).
