# Supplementary material for: The hierarchy of root branching order determines bacterial composition, microbial carrying capacity and microbial filtering
Source: Commun Biol. 2021 Apr 19;4:483. doi: 10.1038/s42003-021-01988-4 (PMC8055976; doi:10.1038/s42003-021-01988-4)
Supplement: Supplementary file 2 — Reporting Summary [file 42003_2021_1988_MOESM2_ESM.pdf]

nature research

Corresponding author(s): Prof. David M. Eissenstat and Prof. Terrence H. Bell

Last updated by author(s): Feb 24, 2021

Reporting Summary

Nature Research wishes to improve the reproducibility of the work that we publish. This form provides structure for consistency and transparency in reporting. For further information on Nature Research policies, see our Editorial Policies and the Editorial Policy Checklist.

Statistics

For all statistical analyses, confirm that the following items are present in the figure legend, table legend, main text, or Methods section.

n/a	Confirmed	
☐	☒	The exact sample size (*n*) for each experimental group/condition, given as a discrete number and unit of measurement
☐	☒	A statement on whether measurements were taken from distinct samples or whether the same sample was measured repeatedly
☐	☒	The statistical test(s) used AND whether they are one- or two-sided<br>*Only common tests should be described solely by name; describe more complex techniques in the Methods section.*
☐	☒	A description of all covariates tested
☐	☒	A description of any assumptions or corrections, such as tests of normality and adjustment for multiple comparisons
☐	☒	A full description of the statistical parameters including central tendency (e.g. means) or other basic estimates (e.g. regression coefficient) AND variation (e.g. standard deviation) or associated estimates of uncertainty (e.g. confidence intervals)
☐	☒	For null hypothesis testing, the test statistic (e.g. *F*, *t*, *r*) with confidence intervals, effect sizes, degrees of freedom and *P* value noted<br>*Give P values as exact values whenever suitable.*
☒	☐	For Bayesian analysis, information on the choice of priors and Markov chain Monte Carlo settings
☒	☐	For hierarchical and complex designs, identification of the appropriate level for tests and full reporting of outcomes
☒	☐	Estimates of effect sizes (e.g. Cohen's *d*, Pearson's *r*), indicating how they were calculated

Our web collection on statistics for biologists contains articles on many of the points above.

Software and code

Policy information about availability of computer code

Data collection	No software was used for data collection
Data analysis	Sequencing data were analyzed with Mothur and QIIME. Processed sequencing data were analyzed in the R statistical environment with the vegan, phyloseq and stats packages.

For manuscripts utilizing custom algorithms or software that are central to the research but not yet described in published literature, software must be made available to editors and reviewers. We strongly encourage code deposition in a community repository (e.g. GitHub). See the Nature Research guidelines for submitting code & software for further information.

Data

Policy information about availability of data

All manuscripts must include a data availability statement. This statement should provide the following information, where applicable:

- Accession codes, unique identifiers, or web links for publicly available datasets
- A list of figures that have associated raw data
- A description of any restrictions on data availability

Raw data files in FASTQ format were deposited in the NCBI sequence read archive under Bioproject number PRJNA639455

Field-specific reporting

Please select the one below that is the best fit for your research. If you are not sure, read the appropriate sections before making your selection.

☐ Life sciences ☐ Behavioural & social sciences ☒ Ecological, evolutionary & environmental sciences

For a reference copy of the document with all sections, see nature.com/documents/nr-reporting-summary-flat.pdf

Ecological, evolutionary & environmental sciences study design

All studies must disclose on these points even when the disclosure is negative.

Study description	We sought to assess the influence of fine root order on bacterial recruitment, microbial filtering and carrying capacity. The common garden planting contains 18 different tree species which were planted as 1-year old seedlings in 1996 and was constructed with a randomized complete block design. We chose six tree species and sampled two root clusters per tree species at each of the six blocks. Root clusters were separated according to the topological approach (R12, R3 and R45) and six replicates of soil and each root order was collected per tree. In total, we collected 108 root samples and 36 bulk soil samples.
Research sample	Fine roots from six different temperate tree species, including three AM-associating (Acer saccharum, Juglans nigra and Liriodendron tulipifera) and three EM-associating (Carya glabra, Quercus rubra and Pinus strobus) tree species. Fine roots were separated according to functionality, specifically into absorptive, transitional and transportive fine roots. Bulk soil was also collected per tree species.
Sampling strategy	Because of the laborious nature of fine root sampling and separation, our sampling rationale was finding a balance between maximizing replicate (blocks) and time to carefully separate roots by order. We decided on sampling 6 of the 8 blocks with the available man power.
Data collection	Data was collected in the field by co-author Jing Guo
Timing and spatial scale	Samples for subsequent sequencing were collected on two different dates because of the labor intensive nature of collecting and sorting fine roots. These dates were in 2018 on July 3rd and July 13th.
Data exclusions	Two samples were excluded after rarefying the sequencing data because of low counts (38 and 191 counts)
Reproducibility	Since roots were collected from within a common garden forest with consistent management, additional collections of similar material would be very feasible. Homogenization of root segments from multiple locations within a root cluster and multiple root clusters within a plot reduced variability due to small-scale spatial effects, and again increases the chances of detecting the same signals through future sampling. Sequencing preparation and execution were performed using well-established protocols, as was downstream data processing. Sequencing data are freely available, which allows others to reproduce our analyses.
Randomization	Samples were grouped according to tree species and then further grouped according to fine root functionality.
Blinding	For actual sample collection, blinding was not possible, since precise separation of root segments was essential. During amplification for sequencing and initial data processing, sample names were not recoded to avoid any unintentional biases

Did the study involve field work? ☒ Yes ☐ No

Field work, collection and transport

Field conditions	It was sunny on the days of collection for roots used in sequencing (high of 31C and 28C on the collection dates) and all work was performed in the shade. After separating roots, samples were placed directly in Falcon tubes and placed on ice in a cooler. Collected samples were taken to a freezer at our field site every 2 hours.
Location	The experimental site was a common garden forest located at the Russell E. Larson Agricultural Research Center in central Pennsylvania (40°42′N, 77°57′W)
Access & import/export	This study was performed at a common garden forest site that is owned and operated by Penn State. We have full permission to perform manipulations and collections at this site.
Disturbance	The site used for this study is freely available for manipulations and sampling, but we aimed to minimize disturbance in order to maximize the potential for future experiments at this site. Only two root clusters were removed within each plot, and sampling locations were refilled with soil.

Reporting for specific materials, systems and methods

We require information from authors about some types of materials, experimental systems and methods used in many studies. Here, indicate whether each material, system or method listed is relevant to your study. If you are not sure if a list item applies to your research, read the appropriate section before selecting a response.

Materials & experimental systems

n/a	Involved in the study
☒	☐ Antibodies
☒	☐ Eukaryotic cell lines
☒	☐ Palaeontology and archaeology
☒	☐ Animals and other organisms
☒	☐ Human research participants
☒	☐ Clinical data
☒	☐ Dual use research of concern

Methods

n/a	Involved in the study
☒	☐ ChIP-seq
☐	☒ Flow cytometry
☒	☐ MRI-based neuroimaging

Flow Cytometry

Plots

Confirm that:

☐ The axis labels state the marker and fluorochrome used (e.g. CD4-FITC).

☐ The axis scales are clearly visible. Include numbers along axes only for bottom left plot of group (a 'group' is an analysis of identical markers).

☐ All plots are contour plots with outliers or pseudocolor plots.

☒ A numerical value for number of cells or percentage (with statistics) is provided.

Methodology

Sample preparation	Fine roots were collected, immersed in sterile NaCl and subsequently sonicated. This solution was centrifuged to remove larger soil particles and then filtered through a 40 micron filter. An aliquot was taken and stained with SYBR green and the cells were then quantified.
Instrument	MACSQuant Vyb flow cytometer (Miltenyi)
Software	MACSQuant Vyb flow cytometer (Miltenyi) - MACSQuantify Software
Cell population abundance	Cell sorting was not performed
Gating strategy	A negative non-fluorescent sample was run to set voltages on the detectors and to set the gates

☐ Tick this box to confirm that a figure exemplifying the gating strategy is provided in the Supplementary Information.
